# O.6.2-6 Life skills development and transfer in community sports programs

**DOI:** 10.1093/eurpub/ckad133.276

**Published:** 2023-09-11

**Authors:** Güven Alarslan, Liselot ter Harmsel-Nieuwenhuis, Kirsten Verkooijen, Ivo van Hilvoorde, Maria Koelen

**Affiliations:** Chairgroup Health and Society, Wageningen University and Research, The Netherlands; Lectoraat Bewegen, School en Sport, Hogeschool Windesheim, The Netherlands; guven.alarslan@wur.nl; Chairgroup Health and Society, Wageningen University and Research, The Netherlands; Lectoraat Bewegen, School en Sport, Hogeschool Windesheim, The Netherlands; guven.alarslan@wur.nl; Chairgroup Health and Society, Wageningen University and Research, The Netherlands

## Abstract

**Purpose:**

Participating in community sports programs can contribute to positive developmental outcomes, such as the development and transfer of life skills. However, studies specifically focusing on which life skills are developed and whether these skills are indeed transferred to other life domains among socially vulnerable adults, are still lacking. Therefore, the aim of this study was to investigate life skills development and transfer among socially vulnerable adults as a result of pariticpating in in community sports programs.

**Methods:**

This study builds on a life-course approach. Data were collected through semi-structured interviews with a total of 13 program participants and three social sports coaches. Additionally, four focus group discussions were organized in which a total of four program coordinators, five social sports coaches and six social workers participated. By employing multiple methods and data sources, data triangulation was achieved. Data were analyzed using thematic analysis.

**Results:**

We found that program participants developed cognitive, emotional, and social life skills. Examples of cognitive life skills were planning and time management, taking initiative, critical thinking, self-awareness, and self-direction. Examples of emotional life skills were coping skills, empathy, and staying mentally positive. Examples of social skills were expressing feelings, assertiveness, leadership, respect, and teamwork. Although the views of the program participants, their coaches and the results from the focus group discussions showed overlap, differences in which life skills were developed and/or transferred by program participants were also found. For example, the development and transfer of planning and time management was mentioned by several social sports coaches and during the focus group discussions, however, this was not always mentioned by the program participants.

**Conclusions:**

Adults in a socially vulnerable position (can) develop cognitive, emotional and social life skills by participating in community sports programs. Moreover, the transfer of these life skills to other life domains like home and work was demonstrated. These findings are an important step towards better understanding the societal impact of community sports programs serving socially vulnerable adults.

